# Coronary stent restenosis and the association with allergy to metal content of 316L stainless steel

**DOI:** 10.5830/CVJA-2017-036

**Published:** 2018

**Authors:** Slodownik D, Swaid F, Ingber A, Danenberg C, Merkin D, Lotan H, Durst R, Moshe S

**Affiliations:** Department of Dermatology, Hadassah Hebrew University Medical Centre, Jerusalem, Israel; Department of Dermatology, Hadassah Hebrew University Medical Centre, Jerusalem, Israel; Department of Dermatology, Hadassah Hebrew University Medical Centre, Jerusalem, Israel; Cardiology Division, Hadassah Hebrew University Medical Centre, Jerusalem, Israel; Cardiology Division, Hadassah Hebrew University Medical Centre, Jerusalem, Israel; Cardiology Division, Hadassah Hebrew University Medical Centre, Jerusalem, Israel; Cardiology Division, Hadassah Hebrew University Medical Centre, Jerusalem, Israel; Sackler Faculty of Medicine, School of Public Health, Department of Environmental and Occupational Health, Tel Aviv University, Tel Aviv, Israel

**Keywords:** stent restenosis, metal allergy, stainless steel

## Abstract

**Background:**

Most intra-coronary stents in use are made of 316 L stainless steel, which contains nickel, chromate and molybdenum. Whether inflammatory and allergic reactions to metals contribute to in-stent restenosis is still a matter of debate.

**Aim:**

The aim of this study was to ascertain the relationship between metal allergy and the occurrence of in-stent restenosis.

**Methods:**

Ninety-nine adult patients who underwent two cardiac catheterisations, up to two years apart, were included in the study. Seventy patients had patent stents at the second angiogram (patent stent group) and 29 were found to have in-stent restenosis (restenosis group). All patients underwent patch testing with the relevant metals and the 316L stainless steel plate.

**Results:**

Twenty-eight (28.3%) patients were found to have an allergy to at least one metal. There was no significant difference in the prevalence of metal allergy between the patent stent group and the restenosis group (28.6 and 27.6%, respectively; p = 0.921).

**Conclusion:**

Our data do not support the theory that contact allergy plays a role in the pathogenesis of in-stent restenosis.

Risk factors for in-stent restenosis, such as diabetes mellitus, diameter of the treated artery, length of the lesion and localisation are well known. In-stent restenosis (ISR) results from excessive fibroproliferative and inflammatory responses to the insult on the arterial wall, leading to neo-intimal proliferation.

Hypersensitivity reaction to metals may be part of the inflammatory process and one of the triggering factors in ISR.^1^ Contact allergy is a common health concern worldwide, with an estimated 15 to 20% of Western populations being hypersensitive to at least one metal allergen.^2^ Recently, much progress has been made regarding the mechanisms underlying inflammatory responses to this unique group of contact allergens, including innate immune activation and T-cell activation by common metal allergens, such as nickel, cobalt, palladium and chromate.^3^

Koster and co-workers^1^ were the first to demonstrate a higher incidence of ISR in patients with delayed hypersensitivity to metals, especially to nickel and molybdenum. Two years later, Hillen et al.^4^ published a study that showed no significant differences in the incidence of restenosis in patients with hypersensitivity to metals, compared to patients without hypersensitivity to metals. Similarly, Iijima^5^ demonstrated that metal allergy was not associated with restenosis after initial stent implantation. However, metal allergy was frequently observed in patients with ISR recurrence.

Given the impact of ISR on coronary patient morbidity and mortality rates, and given the contradictory data available in the current literature, we conducted a case–control study aimed at identifying an association between metal allergy and ISR.

## Methods

An informed, written consent was obtained from all patients. The study received the approval of the local institutional review board for human research.

Ninety-nine patients aged 18 years and older, who underwent at least two coronary artery catheterisations within a period of two years at the Department of Cardiology, Hadassah University Hospital in Jerusalem, were enrolled into the study. A bare-metal stent was implanted in one coronary vessel during the first catheterisation. The second catheterisation was performed to assess the degree of restenosis.

Catheterisation was performed, using the Seldinger technique, through the femoral artery with 6F standard catheters. In case of intervention, a guiding catheter was introduced over a wire. Stent implantation was usually performed after balloon predilatation.

Patients were divided into two groups as follows. The study group consisted of 29 patients who underwent implantation of at least one stent at the first catheterisation and were found to have ISR during the second catheterisation. The control group consisted of 70 patients who underwent implantation of at least one stent at the first catheterisation and were found to have a patent lumen during the second catheterisation.

The presence or absence of ISR was determined by the cardiologists who performed the catheterisation. Significant stenosis was defined as stenosis of 50% or more of the coronary lumen. Table 1 shows the patients’ characteristics. All stents were made of 316L stainless steel.

**Table 1:** Characteristics of the study and control group individuals

*Characteristic*	*Study group (n = 29)*	*Control group (n = 70)*	*p-value*
Age	64.7 ± 7.2	62.9 ± 5.8	0.6003
Diabetes	11 (38)	27 (39)	0.827
Hypertension	14 (48)	31 (44)	0.713
Smoking	16 (55)	34 (49)	0.557
Female	8 (28)	17 (24)	0.689
Hyperlipidaemia	14 (48)	36 (51)	0.778

Exclusion criteria included insertion of drug-eluting stents, immunosuppressive therapy, pregnancy, and marked cutaneous inflammation, especially at the patch testing site. Neither the study nor the control group included HIV-infected patients.

Three patients in the study group and five in the control group had a prior history of metal allergies. All patients were tested with the metals listed in Table 2. They were patch tested using allergens from Chemotechnique Diagnostics^®^ (Malmö, Sweden). Patches were applied onto the patient’s upper back using Finn Chambers^®^ on Scanpor^®^ (Epitest OY, Tuusula, Finland). All patients were tested for reactions, which were read at D2/3 and D4/5 using ICDRG criteria.^6^

**Table 2:** Patch-tested metals in the study

*Material*	*Concentration (%)*	*Vehicle*
Nickel sulphate	5	Petrolatum
Potassium dichromate	0.5	Petrolatum
Molybdenum chloride	0.25	Petrolatum
Molybdenum chloride	0.5	Petrolatum
Manganese oxide	10	Petrolatum
Cobalt chloride	1	Petrolatum
316L stainless steel		as is

We used the chi-squared test in order to determine differences between the groups. Statistical significance was determined at a value of p ≤ 0.05.

## Results

The two study groups did not display any significant differences in terms of age, gender, diabetes, hypertension, lipid profiles and smoking status (Table 1). The data from the patch test reactions are provided in Table 3. Of the 99 patients included in the study, 28 (28.3%) had at least one reaction to the tested metals.

**Table 3:** Distribution of positive reactions

*Metal*	*No of positive Metal reactions*	*% of total positive Reactions*	*% of study and control groups*
Nickel	15	37.5	15.1
Chromate	8	20	8
Cobalt	5	12.5	5
Manganese	2	5	2
Molybdenum 0.25%	4	10	4
Molybdenum 0.5%	5	12.5	5
Stainless steel	1	2.5	1
Total positive reactions	40	100	40.1

There were a total of 40 positive reactions in both groups. The most common reactions were to nickel, followed by chromate and cobalt. Four of the patients had reactions to both nickel and cobalt. Table 4 shows the distribution of the positive reactions to metals among the two study groups. Metal sensitivity rate between the two groups did not differ significantly (p = 0.921).

**Table 4:** Distribution of metal sensitivity in both study groups

*Metal*	*Study group Metal (n = 29)*	*Control group (n = 70)*	*p-value*
Nickel	5	10	0.641
Chromate	2	6	0.383
Cobalt	2	3	0.491
Manganese	0	2	0.553
Molybdenum 0.25%	1	3	0.935
Molybdenum 0.5%	2	3	0.491
Stainless steel	1	0	0.172
Total positive reactions	13	27	0.556

## Discussion

Grade 316 is the standard molybdenum-bearing grade, second in overall volume production to grade 304 among the austenitic stainless steels. The molybdenum gives grade 316 better overall corrosion-resistant properties than grade 304, particularly higher resistance to pitting and crevice corrosion in chloride environments. Grade 316L, the low-carbon version of 316, has high resistance to sensitisation.^7^

Over a decade has passed since Koster suggested metal allergy may play a role in the pathogenesis of ISR. Follow up on studies^4^,^5^,^8^,^9^ of ISR in patients who had received stainless steel stents did not confirm Koster’s initial observations.

A recent report from Turkey showed a correlation between nickel allergy and ISR among patients who were treated with cobalt chromium stents, which have a three times higher concentration of nickel than 316L stainless steel stents.^10^ It has been speculated that nickel ions may influence expression of the adhesion molecule ICAM-1 in endothelial cells,^11^ which in turn may trigger local inflammation and lead to ISR. By contrast, Thyssen et al.^12^ studied a large cohort of patients with pre-existing nickel allergy and found that these individuals did not appear to have a higher risk for ISR.

In comparison with the above studies, both our study and control groups had higher positive reaction rates to nickel and chromate. This comes as no surprise as metal allergy is more common in Israel,^13^,^14^ compared to Europe and North America.^15^,^16^ Legislative and market-related factors result in higher metal sensitisation rates in Israel. There were no significant differences, however, between our study and control groups.

Our results are in line with most earlier studies and do not support a role for nickel, cobalt, chromate or molybdenum allergy in ISR. Conversely, recent convincing data demonstrate that gold allergy is a contributing factor to ISR. It is possible that gold, which is a more potent sensitiser than nickel,^17^ may induce a stronger immunological reaction, resulting in endothelial proliferation.

The weaknesses of our study, as of previously published reports, are its relatively small size and its retrospective design. We suggest the need for larger, prospective, confirmatory cohort studies of patients with ISR.

## Conclusion

Our data do not support the role of contact allergy in the pathogenesis of in-stent restenosis.
